# Agenesis of the right lung in an adult woman: A case report

**DOI:** 10.1002/ccr3.8107

**Published:** 2023-10-19

**Authors:** Elias Gonçalves, Ofélia Sachicola, Bartolomeu Estanislau, Francisca Quifica, Humberto Morais, Margarete Arrais

**Affiliations:** ^1^ Serviço de Pneumologia Complexo Hospitalar de Doenças Cardiopulmonares, Cardeal Dom Alexandre do Nascimento Luanda Angola; ^2^ Centro de Estudos Avançados em Educação e Formação Médica, Faculdade de Medicina Agostinho Neto University Luanda Angola; ^3^ Departamento de Cardiologia Hospital Militar Principal/Instituto Superior Luanda Angola; ^4^ Department of Pulmonology Military Hospital Luanda Luanda Angola; ^5^ Centro de Investigação em Saúde de Angola (CISA) Caxito Bengo Angola

**Keywords:** agenesis, bronchofibroscopy, computed tomography, right lung

## Abstract

**Key Clinical Message:**

Pulmonary agenesis is a rare congenital abnormality. Patients with hemithorax white‐out on x‐rays should be treated with caution, especially in resource‐limited countries where chest CT and bronchofibroscopy are not available to confirm the diagnosis.

**Abstract:**

Pulmonary agenesis is an uncommon congenital abnormality defined by the complete absence of the lung parenchyma, as well as the bronchial and vascular structures. Right‐sided pulmonary agenesis is less frequent, has a worse prognosis, and is usually associated with other congenital abnormalities. We reported the clinical case of a 31‐year‐old woman with right pulmonary agenesis, and no other congenital abnormalities, whose diagnosis was confirmed by thoracic computed tomography and bronchofibroscopy and who has a good prognosis.

## Graphical abstract

The gold standard for identifying pulmonary agenesis is a chest CT scans and bronchofibroscopy.

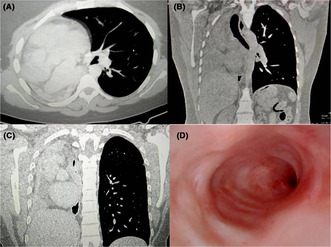

## 1 INTRODUCTION

Congenital pulmonary abnormalities represent a group of illnesses that are often detected in the prenatal period and in childhood, with adulthood being extremely rare.^1^ Development lung abnormalities can be divided into pulmonary agenesis when there is a total absence of the lung parenchyma, bronchi, and pulmonary artery; pulmonary aplasia when there is an absence of the lung and pulmonary artery, but with the presence of rudimentary bronchi; and pulmonary hypoplasia when there is a hypoplasia of the bronchi and pulmonary artery, with variable lung parenchyma.^2^ Bilateral agenesis and hypoplasia are incompatible with life.^2^ Other congenital abnormalities such as cardiovascular, musculoskeletal, gastrointestinal, and genitourinary, are related in more than 50% of the cases, and when this happens the diagnosis is early.^2^ Adulthood diagnosis is delayed due to the absence of other congenital abnormalities.^2^ When asymptomatic the diagnosis is usually accidental through chest imaging tests, but when symptomatic, they are frequently misdiagnosed as pulmonary collapse or pleural effusion.^3^ When the involved side is the left, they are more common (about 70%) and have a better prognosis, than when the involved side is the right.^3^ The prognosis depends on the extent of the anomalies and the presence of other congenital abnormalities.^4^ We describe the case of a woman who was diagnosed with right pulmonary agenesis at the age of 31, and who has a good prognosis.

## 2 CASE REPORT

This is a 31‐year‐old woman from a city in the interior of Angola who was seen in an outpatient pulmonology clinic in March 2023, at the Complexo Hospitalar de Doenças Cardiopulmonares (CHDCP) in Luanda, the capital city of Angola, for recurrent episodes of moderate intensity and exertion pain in the right hemithorax without irradiation and relieved with analgesics, for more than 10 years. There was no history of cough, expectoration, dyspnea, fever, anorexia, or weight loss in our patient.

She has a history of HIV infection and has been on antiretroviral therapy since she was 19 years old. A chest x‐ray revealed destruction of the right lung at the time of diagnosis, which was interpreted as tuberculosis. For this reason, despite negative tuberculosis tests, complete treatment for pulmonary tuberculosis was recommended. She returned to the hospital at the age of 29 with pain in her right hemithorax, and because the radiological image persisted, tuberculosis therapy was recommended once more. She had another chest x‐ray in 2023 (at the age of 31) and was referred to a pulmonologist in Luanda, since the loss of volume in her right lung persisted. She had no smoking or alcoholism history, and no knowledge of congenital disorders or other personal or family histories. Our patient has four children from normal pregnancies, and she was already on antiretroviral therapy during her past two pregnancies. The oldest child is 15 years old, and the youngest is 4 years old; all are in good health.

On physical examination, she appears to be in reasonable general and nutritional condition, with slightly hypo‐colored mucous membranes. She was eupneic and acyanotic, with no jugular engorgement or lymphadenopathy. Vital signs within normal parameters (HR: 69/min; BP:112/68 mmHg; SpO_2_: 96% at ambient air). Thorax without scars, collateral venous circulation but, with reduced respiratory movement, reduced thoracovocal fremitus and dullness to percussion in the right hemithorax. Absence of vesicular breath sound in the right hemithorax, without adventitious sounds. Heart sounds (S1 and S2) audible in the region of the xiphoid appendix and in the second intercostal space, in the right parasternal line, rhythmic and regular, without murmurs or extra sounds. The abdomen was flat, mobile with respiratory movements, soft, without pain and without visceromegaly. Lower limbs without edema.

**FIGURE 1:**
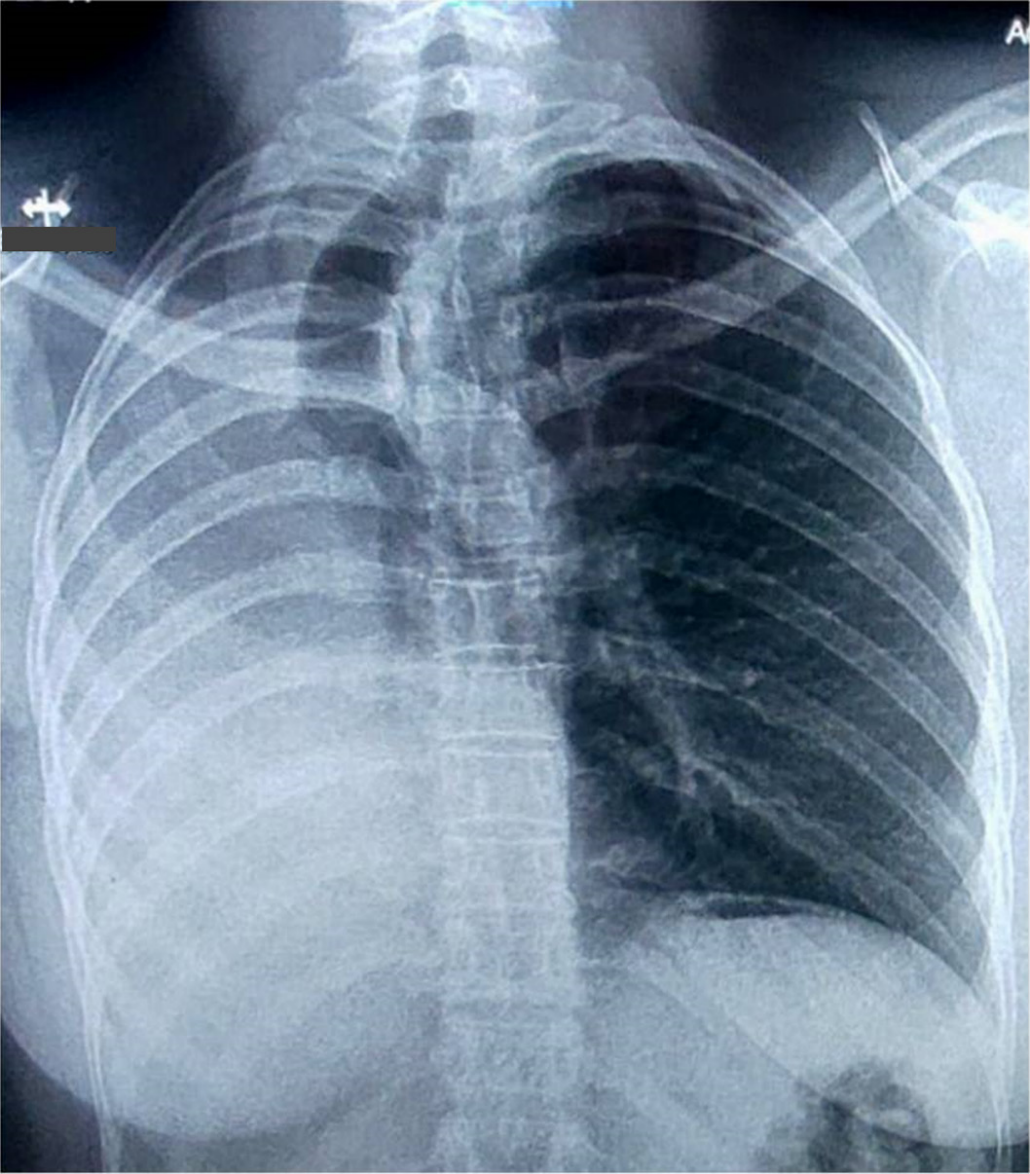
Posteroanterior chest x‐ray: Lung volume loss on the right hemithorax, obliterating the right costo and cardio‐phrenic angle, with the displacement of the mediastinal structures to the right, and absence of the right main bronchus.

**FIGURE 2:**
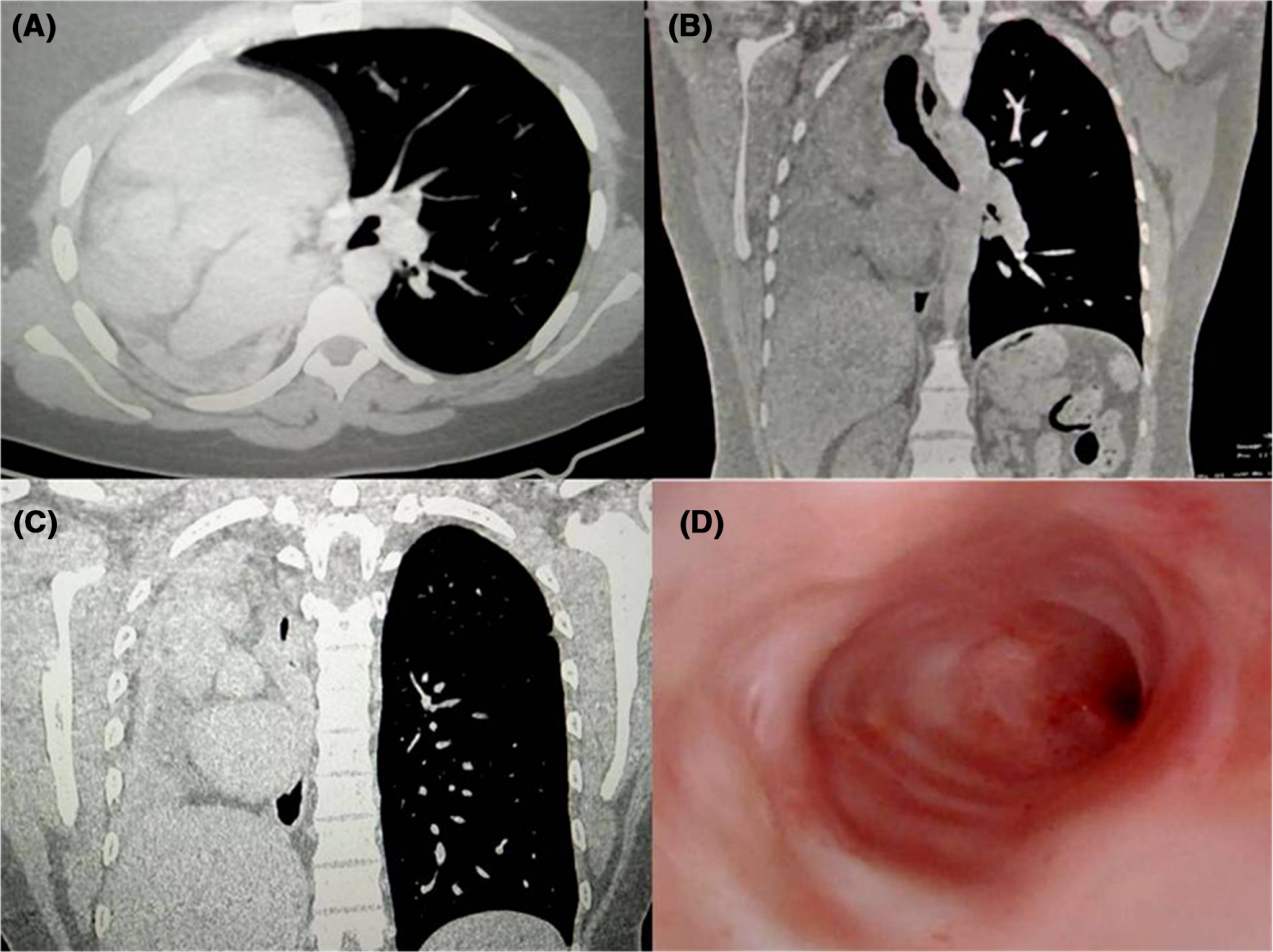
(A–C) Chest computed tomography, axial (A), and coronal (B and C) planes: Absence of right lung parenchyma, bronchial and vascular structures, with right‐sided herniation and displacement of the mediastinal structures to the right. Bronchofibroscopy (D): Absence of the carina and right main bronchus, with preserved left bronchial tree structures.

A posteroanterior (PA) chest radiograph revealed lung volume loss on the right hemithorax, obliteration of the costo and cardio‐phrenic angles, mediastinal structures displaced to the right, and the absence of the right main bronchus (Figure 1). Thoracic computed tomography confirmed the complete absence of the right lung parenchyma, the bronchial and vascular structures, as well as right‐sided herniation and mediastinal structures displacement to the right (Figure 2A–C). Bronchofibroscopy revealed the absence of carina and right main bronchus, with preserved structures of the left bronchial tree (Figure 2D). Electrocardiogram showed a sinus rhythm, and a heart rate of 60 cycles per minute. Poor progression of the R wave from V1 to V6. Nonspecific changes in ventricular repolarization were observed, and a transthoracic echocardiogram revealed normal aortic root and cardiac cavities dimensions, as well as a left ventricular ejection fraction of 65%. Mild pulmonary arterial hypertension (systolic pressure in the pulmonary artery = 36 mmHg), without other anomalies. Ultrasonography and abdominal tomography did not reveal any abnormalities.

The patient was informed about her diagnosis and has been followed regularly in the outpatient pulmonology clinic, maintaining the treatment and monitoring of HIV infection by an infectious diseases physician.

## 3 DISCUSSION

Pulmonary agenesis is a rare congenital abnormality whose etiology is not well known.^2^ It is believed to be due to certain genetic factors, environmental factors, vitamin A or folate deficiency, and viral infections.^5^, ^6^

It is usually diagnosed in the prenatal period and in early childhood,^2^ is more common on the left, and has a better prognosis than right‐sided pulmonary agenesis.^3^ In most cases of pulmonary agenesis, other congenital abnormalities are associated, and when this happens the prognosis is worse.

We present the case of a 31‐year‐old woman who has right‐sided pulmonary agenesis, with only recurrent pain in the right hemithorax as her main complaint. Probably due to her HIV infection and the persistence of lung volume loss on the right on the chest x‐ray, she was treated on two occasions for pulmonary tuberculosis, despite negative tuberculosis tests. Our patient was treated in a city in the interior of Angola, where patients with mild symptoms and tuberculosis suspicion are followed by trained nurses, who are not always able to correctly interpret chest x‐rays and make the appropriate differential diagnosis. Patients in these regions are only referred to a doctor if their tuberculosis has not resolved. For this reason, the patient was misdiagnosed for approximately 12 years.

When patients are asymptomatic or have mild, recurring symptoms, they represent a major challenge in differential diagnosis, particularly in countries with limited resources, where specific diagnostic tests such as computed tomography and bronchofibroscopy are not available. As a result, these patients are diagnosed and treated for the most frequent lung diseases that cause ipsilateral lung volume loss.

Our patient was diagnosed in adulthood, and she has no other congenital abnormalities, which makes it a rare case with a better prognosis. Other cases with the same illness have been reported, and the diagnosis was established accidentally during routine imaging tests^7^ or during the diagnostic assessment of pulmonary symptoms.^8^, ^9^, ^10^, ^11^

In pulmonary agenesis, diagnosis is usually made using chest imaging tests. The most common radiographic findings are ipsilateral lung volume loss, obliteration of the costal and cardiophrenic angles, displacement of the mediastinal structures toward the affected side, the elevation of the ipsilateral hemidiaphragm, and compensatory hyperinflation of the contralateral lung.^12^

It is important to make a differential diagnosis with pathologies that have similar findings, such as acquired lung collapse, pleural thickening, pneumoectomy, pulmonary destruction, pleural effusion, lung cancer, diaphragmatic hernia, cystic adenomatoid malformation, pulmonary hypoplasia, and pulmonary sequestration.^8^, ^12^

Chest CT scans and bronchofibroscopy provide detailed information about the bronchial tree, parenchyma, and pulmonary vessels and are considered the gold standard for diagnosis.^13^ In our patient, the diagnosis was confirmed by chest CT scans and bronchofibroscopy, which showed the absence of the right lung parenchyma, as well as its bronchial and vascular structures.

Asymptomatic patients, with mild symptoms and no other congenital abnormalities, do not require treatment; however, lung infections or other respiratory diseases should be treated early and the patient should have clinical follow‐up to detect possible complications, such as pulmonary hypertension,^8^, ^11^, ^14^ which was recommended for our patient, who had mild pulmonary hypertension on echocardiogram. In circumstances where additional congenital abnormality is present, surgical treatment may be required.^8^, ^14^

## 4 CONCLUSION

Unilateral pulmonary agenesis is a rare congenital abnormality that should be part of the differential diagnosis of every patient presenting with a chest x‐ray with unilateral persistent lung volume loss, with the absence of the ipsilateral bronchus. Early diagnosis prevents complications and unnecessary treatments, and improves survival.

## AUTHOR CONTRIBUTIONS

**Elias Gonçalves:** Data curation; investigation; writing – original draft. **Ofélia Sachicola:** Data curation; investigation; writing – original draft. **Bartolomeu Estanislau:** Data curation; investigation. **Francisca Quifica:** Investigation; writing – original draft. **Humberto Morais:** Conceptualization; supervision; writing – review and editing. **Margarete Arrais:** Conceptualization; supervision; writing – original draft; writing – review and editing.

## FUNDING INFORMATION

No source of funding.

## CONFLICT OF INTEREST STATEMENT

The authors do not have any financial and nonfinancial competing interests.

## ETHICAL APPROVAL

This manuscript was completed in accordance with the ethical standards of the institutional Ethics Committee, and the patient signed consent for publication.

## CONSENT

Written informed consent was obtained from the patient to publish this report, including images, in accordance with the journal's patient consent policy.

## Data Availability

The data that support the findings of this study are available from the corresponding author upon reasonable request.

## References

[1] Cherian SV , Kumar A , Ocazionez D , Estrada‐Y‐Martin RM , Restrepo CS . Developmental lung anomalies in adults: a pictorial review. Respir Med. 2019;155:86‐96. doi:10.1016/j.rmed.2019.07.011 31326738

[2] Berrocal T , Madrid C , Novo S , Gutiérrez J , Arjonilla A , Gómez‐León N . Congenital anomalies of the tracheobronchial tree, lung, and mediastinum: embryology, radiology, and pathology. Radiographics. 2004;24(1):e17. doi:10.1148/rg.e17 14610245

[3] Kumar P , Tansir G , Sasmal G , Dixit J , Sahoo R . Left pulmonary agenesis with right lung bronchiectasis in an adult. J Clin Diagn Res. 2016;10(9):OD15‐OD17. doi:10.7860/JCDR/2016/21623.8547 PMC5072001 27790501

[4] Lee EY , Dorkin H , Vargas SO . Congenital pulmonary malformations in pediatric patients: review and update on etiology, classification, and imaging findings. Radiol Clin North Am. 2011;49(5):921‐948. doi:10.1016/j.rcl.2011.06.009 21889015

[5] Currarino G , Williams B . Causes of congenital unilateral pulmonary hypoplasia: a study of 33 cases. Pediatr Radiol. 1985;15(1):15‐24. doi:10.1007/BF02387847 3969292

[6] Roque A , Burton E , Boedy R , Falls G , Bhatia J . Unilateral pulmonary agenesis without mediastinal displacement. South Med J. 1997;90(3):335‐337. doi:10.1097/00007611-199703000-00015 9076309

[7] El‐Badrawy A , El‐Badrawy MK . Adult presentation of asymptomatic right lung agenesis: a rare anatomical variation. Surg Radiol Anat. 2019;41(2):247‐249. doi:10.1007/s00276-018-2130-1 30402711

[8] Roy PP , Datta S , Sarkar A , Das A , Das S . Unilateral pulmonary agenesis presenting in adulthood. Respir Med Case Rep. 2012;5(1):81‐83. doi:10.1016/j.rmedc.2011.05.003 26057001 PMC3920441

[9] Sayit AT , Elmali M . An adult patient presenting with right unilateral pulmonary agenesis: a case report and literature review. Surg Radiol Anat. 2020;42(11):1299‐1301. doi:10.1007/s00276-020-02467-x 32266442

[10] Rahman SMT , Shahriar T , Akhter KM , Tarannum A , Hossain M . A rare variant of right sided pulmonary agenesis presenting in adulthood: 1st reported case from Bangladesh. Respir Med Case Rep. 2022;37:101629. doi:10.1016/j.rmcr.2022.101629 35309975 PMC8927843

[11] Pimenta DA , Aguiar FL , Fernandes BC , Rolo R . Late diagnosis of pulmonary agenesis. BMJ Case Rep. 2021;14(10):e245233. doi:10.1136/bcr-2021-245233 PMC8552127 34706915

[12] Gunbey HP , Gunbey E , Sayit AT , Bulut T . Unilateral right pulmonary agenesis in adulthood. J Clin Diagn Res. 2014;8(6):3‐4. doi:10.7860/JCDR/2014/7968.4420 PMC4129333 25121044

[13] Schwartz MZ , Ramachandran P . Congenital malformations of the lung and mediastinum – a quarter century of experience from a single institution. J Pediatr Surg. 1997;32(1):44‐47. doi:10.1016/S0022-3468(97)90090-7 9021566

[14] Chopra K , Sethi G , Kumar A . Pulmonary agenesis. Indian Pediatr. 1988;25(7):678‐682. 3220546

